# Assessment of podocyte detachment as a pivotal step in the development of focal segmental glomerulosclerosis

**DOI:** 10.1186/s43046-024-00244-0

**Published:** 2024-11-18

**Authors:** Ikbal Ahmed Abdo Elkholy, Wagdi Elkashef, Fatma El-Husseini Mostafa, Amany Hassan

**Affiliations:** Mansoura University, Mansoura, Egypt

**Keywords:** Nephrotic syndrome, Focal segmental glomerulosclerosis, Podocytopathy, Electron microscopy

## Abstract

**Background:**

Podocytopenia refers to a decrease in the number of podocytes. When podocytes are injured, they may detach leading to podocytopenia, which represents a critical step in the development of podocytopathy and subsequently deterioration of renal functions. Pathological assessment of podocytopenia plays a crucial role in diagnosing underlying kidney diseases.

**Aim:**

To assess detached podocytes and evaluate their diagnostic role in the development of focal segmental glomerulosclerosis.

**Materials and methods:**

This is a retrospective study, conducted on 67 archival renal biopsies with the clinical diagnosis of steroid-resistant or steroid-dependent nephrotic syndrome (SRNS) and diagnosed as focal segmental glomerulosclerosis (FSGS) and podocytopathy with detached podocytes by electron microscopy (EM). Colloidal iron stain and Desmin immunohistochemical stain were performed. Assessment of the mean percent of stained pixels in relation to the surface tuft area of the glomerulus, i.e., mean percent of stained area (PSA) was done using image analysis system (ImageJ 1.52a) software.

**Results:**

Podocytopathy with detached podocytes was diagnosed in 35 (52.24%) cases, while FSGS was diagnosed in 32 (47.76%) cases. Regarding detached podocytes, 27 (49.3%) cases showed no detached podocytes by light microscopy (LM), while only 4 (6%) showed severe podocyte detachment. There was a statistically significant difference between control cases and both podocytopathy with detached podocytes and FSGS regarding mean PSA (*p* ≤ 0.001).

**Conclusion:**

Standardized reporting of detached podocyte cells is becoming mandatory as they have a high positive predictive value for the expected EM picture.

## Introduction

Podocytopathy is defined as a group of proteinuric glomerular diseases in which proteinuria is attributed to intrinsic or extrinsic podocyte injury and where the podocyte genotype is altered with damage or dysfunction of podocytes [1, 2]. In podocytopathies, nephrin and podocalyxin appear in urine before proteinuria and microalbuminuria which were previously considered as earliest markers of nephropathies and podocyte injury [3, 4]. These urinary markers are important in early diagnosis of primary and secondary nephropathies [5, 6]. Nephrotic syndrome (NS) is the most common presentation of glomerular diseases in children [7]. It can be congenital or acquired; the acquired causes can either be idiopathic (primary) or secondary [8]. In children, about 20% of cases of NS in children and 40% in adults are due to focal segmental glomerulosclerosis (FSGS) [9]. FSGS accounts for the most prevalent worldwide acquired kidney disease leading to end-stage kidney disease in children and some cases have a tendency to recur in individuals with kidney transplantation [10]. It is not a single disease, but rather a histological pattern of glomerular lesions that share injury within the podocyte as a primary pathophysiological feature [11]. McGrogan et al*.* [12] revised published literature from all over the world and reported that the annual incidence rates for FSGS were about 0.2/100,000 population/year. Australia has ranked the highest incidence of FSGS. In the southwestern USA, FSGS was the most common diagnosis forming about 39% of about 2500 adult kidney biopsies examined between the years 2000 and 2011 [13]. Incidence rates are generally higher in men, being about 1.5-fold higher than in women [14]. FSGS presents with NS and less responsiveness to steroid therapy than minimal change disease (MCD) [15]. Recently, attention has been focused on the role of podocytes in the pathogenesis of NS [16]. The response to podocytes injury as highly differentiated cells is not typical, and once they are damaged, there is no regeneration. They cannot enter the cell cycle and a progression towards glomerulosclerosis occurs [17]. Barisoni’s classification of primary podocytopathy stated that the loss of podocytes is the differentiating feature between MCD and the development of FSGS [18]. Hence, assessment of detached podocytes and evaluation of their diagnostic role in the development of FSGS is becoming urgently important in the last few years.

## Material and methods

This is a retrospective study, conducted on 82 archival renal biopsy specimens collected from the university hospital pathology lab through the period from January 2021 to January 2023 with the clinical diagnosis of SRNS. *Inclusion criteria* include (1) cases diagnosed as FSGS and podocytopathy with detached podocytes by EM examination and (2) available EM results. *Exclusion criteria* include (1) cases diagnosed as MCD by EM. (2) Cases with no available paraffin blocks were seven cases and (3) cases with no remained tissue in paraffin blocks for evaluation were eight cases, so they were excluded from the study. Finally, cases eligible for this study were 67 cases. Ten control cases were obtained from surgical nephrectomy specimens done for Wilm’s tumor or hypernephroma. Clinical data including age and clinical presentation were collected from patients’ medical records. Ethical consent was obtained from patients to perform the study. This study was approved by the Institutional Review Board of the School of Medicine.

This study proceeded in the following steps:

1. *Pathological assessment of renal biopsies:* All studied renal biopsies were obtained and fixed in 10% neutral buffered formalin and then processed for paraffin sections. Slides were stained by hematoxylin and eosin (H&E), and routine renal biopsy stains were performed at our institution (periodic acid-Schiff, Masson trichrome, and periodic acid silver methenamine stains). All the slides have been examined under LM using a Nikon Eclipse 600 microscope (Nikon, Burlingame, CA, USA). While assessing H&E-stained sections, each glomerulus was evaluated for detached podocyte number in Bowman’s space.

### Scoring of detached podocytes by LM

*Score 0:* Absent/No detached podocytes. Score 1 (Mild podocytopathy): 1–2 detached podocytes/glomerulus in < 25% of all glomeruli. *Score 2 (Moderate podocytopathy):* 1–2 detached podocytes/glomerulus in 25–50% or ≥ 50% of all glomeruli. *Score 3 (Severe podocytopathy):* ≥ 3 detached podocytes/glomerulus in > 50% of all glomeruli.

**Fig. 1:**
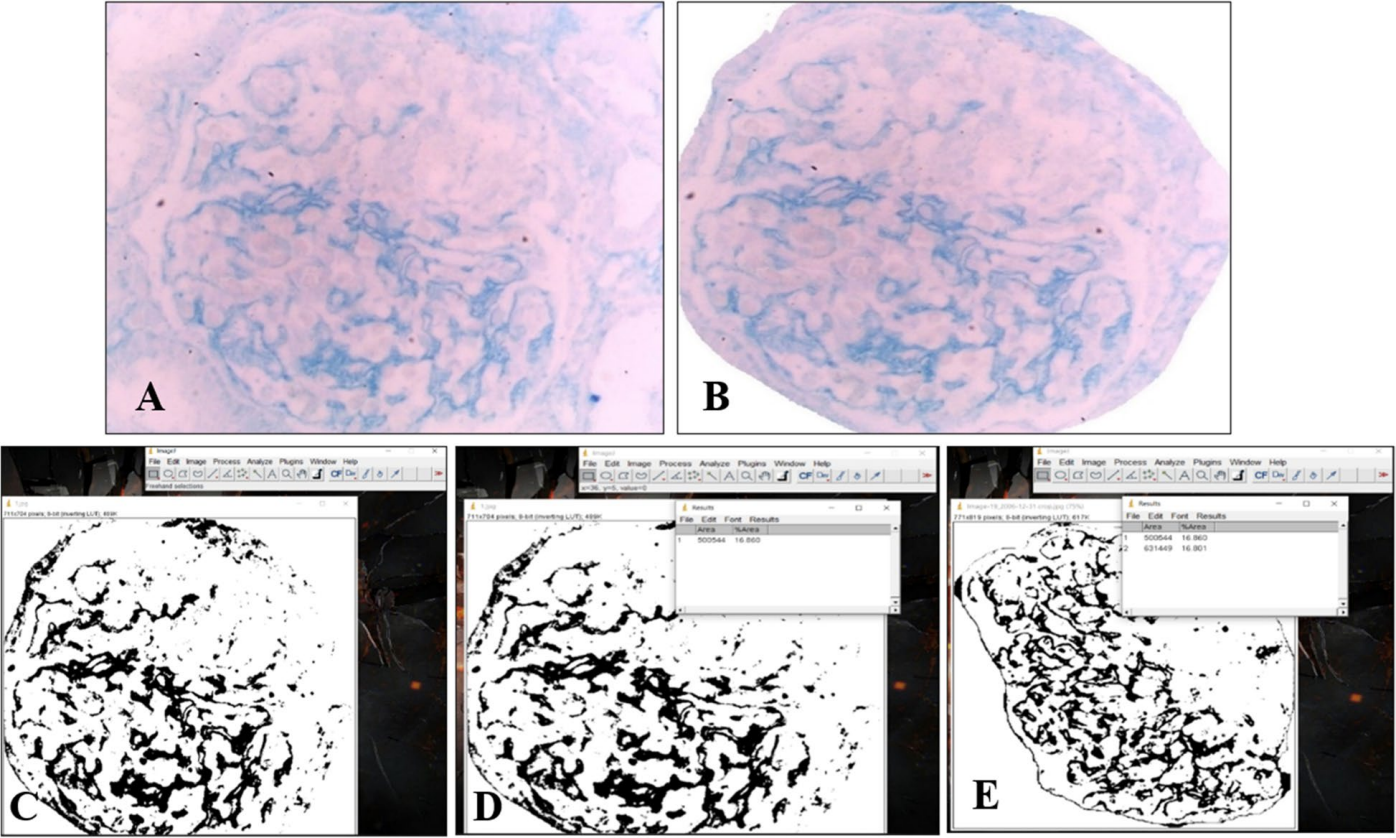
Steps of image analysis by image J software. **A **Each glomerulus in the colloidal iron stained slide is captured separately with the high power maginification; × 40 .The tuft in the upper part of the glomerulus is not stained due to sclerosis. **B **For cropping, the picture is opened in Paint program and cropped along the Bowan’s capsule. **C **The picture is converted to black and white colors, preparing it to work on. **D **The black binary Area (represents the intact capillaries) is obtained in pixels and the percent of black area in relation to the total glomerulus is obtained (PSA). In this example, is 16.860. **E **Results of both two glomeuli are listed together. The results can be summerized and then, the mean of the resultant percentages are calculated to give an idea about the average percent of colloidal iron stain in each case

2. *Colloidal iron special stain:* colloidal iron special stain for the sialomucin coat of podocytes was done for all biopsies to delineate the podocalyxin coat of podocytes. In the staining procedure, the colloidal iron solution has positively charged iron ions that bind with the negatively charged podocalyxin mucin coat of podocytes [19, 20]. Firstly, two non-charged, unstained slides were prepared for each case. Then, the first group of the non-charged slides for all cases were stained. Lastly, the staining process has been repeated again on the second prepared group of unstained slides, to ensure our results.
3. *Desmin immunohistochemical stain:* assessment of podocyte injury was performed via desmin immune stain for all studied biopsies. Immunohistochemical staining was performed on 4-μm thick, formalin-fixed, paraffin-embedded tissue sections. The sections had been deparaffinized then incubated in xylene and hydrated in a series of decreased ethanol concentrations. Heat-induced epitope retrieval was performed using microwave and ethylene diamine tetraacetic acid (EDTA) buffer (PH 9). The sections were washed out in PBS buffer and then immersed in a peroxidase-blocking solution of DAKO to inhibit endogenous peroxidase activity [21]. Immunohistochemical staining of FFPE tissue was achieved using rabbit primary monoclonal antibody against desmin (Cell Marque. Desmin (EP15) ready to use, USA), DAKO kit (Dako REAL™ EnVision™ Detection System, Peroxidase/DAB + , Rabbit/Mouse, Produktionsvej 42, DK-2600, Glostrup, Denmark). The antigen–antibody reaction was highlighted by diamino-benzidine tetrachloride in the chromogen solution. Sections were counterstained with hematoxylin and mounted. Between steps, samples have been carefully washed with PBS [21].
4. EM examination for all cases was performed and correlation with LM results was done.
5. *Calculation of mean percent of stained area (PSA):* In each core biopsy, colloidal iron-stained glomeruli have been examined by two senior independent consultant nephropathologists. Assessment of the mean percent of colloidal iron and desmin-stained pixels in relation to the surface tuft area of the glomerulus; i.e. *mean percent of stained area (PSA)* was accomplished using image analysis system (ImageJ 1.52a) software [22, 23]. This percentage reflects the degree of podocalyxin coat depletion and consequently the degree of podocyte loss. This technique is simple, routine-friendly, objective, and rapid and could be applied to routine core biopsies. The steps have been simplified by being recorded in a macro which permits performing the steps in a few seconds by pressing only one button. Image analysis was performed through a four-step technique [24]. In each core biopsy, individual colloidal iron-stained glomeruli were carefully examined using the same microscope. Every stained glomerulus was photographed with a Nikon DXM 1200 digital camera. Then, each captured photo was examined using the ImageJ program and the PSA was calculated. In the end, the mean value of whole resultant percentages was obtained to give the mean percent of colloidal iron and desmin stained area for each patient (Fig. 1).

### Statistical analysis

Data have been analyzed by the Statistical Package of Social Science (SPSS) program for Windows (Standard version 24). The normality of data has been first tested with a one-sample Kolmogorov–Smirnov test. Qualitative data were described by means of number and percent. Association among categorical variables was tested using the chi-square test while the Monte Carlo test was used when the expected cell count less than 5. Continuous variables were presented as mean ± SD (standard deviation) for normally distributed data and median (Min–Max) for non-normal data. The two groups were correlated by independent *t* test (parametric) and Mann–Whitney test (non-parametric). More than two groups were compared by the ANOVA test (parametric) and Kruskal–Wallis test (non-parametric). Sensitivity and specificity in terms of accuracy at multiple cutoff points were tested by the ROC curve. The results were considered significant when the *p* ≤ 0.05. The smaller the *p* value obtained, the more significant the results.

## Results

The patient group was composed mainly of pediatric age category and male gender forming 64.8% and 72.4% of cases, respectively. Regarding detached podocytes among the patients’ group, 27 (49.3%) cases showed no detached podocytes while only 4 (6%) cases showed severe podocyte detachment as depicted in Table 1.

**Table 1:** Detached podocyte scoring among patients’ group by LM diagnosis

Detached podocytes score by LM	Patients group (no = 67)
Score (0): Absent	27 (40.29%)
Score (1): 1–2/glomerulus in < 25% of total glomeruli	27 (40.29%)
Score (2): 1–2/glomerulus in 25–50% or > 50% of total glomeruli	9 (13.43%)
Score (3): ≥ 3/glomerulus in any percent of total glomeruli	4 (5.97%)

**Fig. 2:**
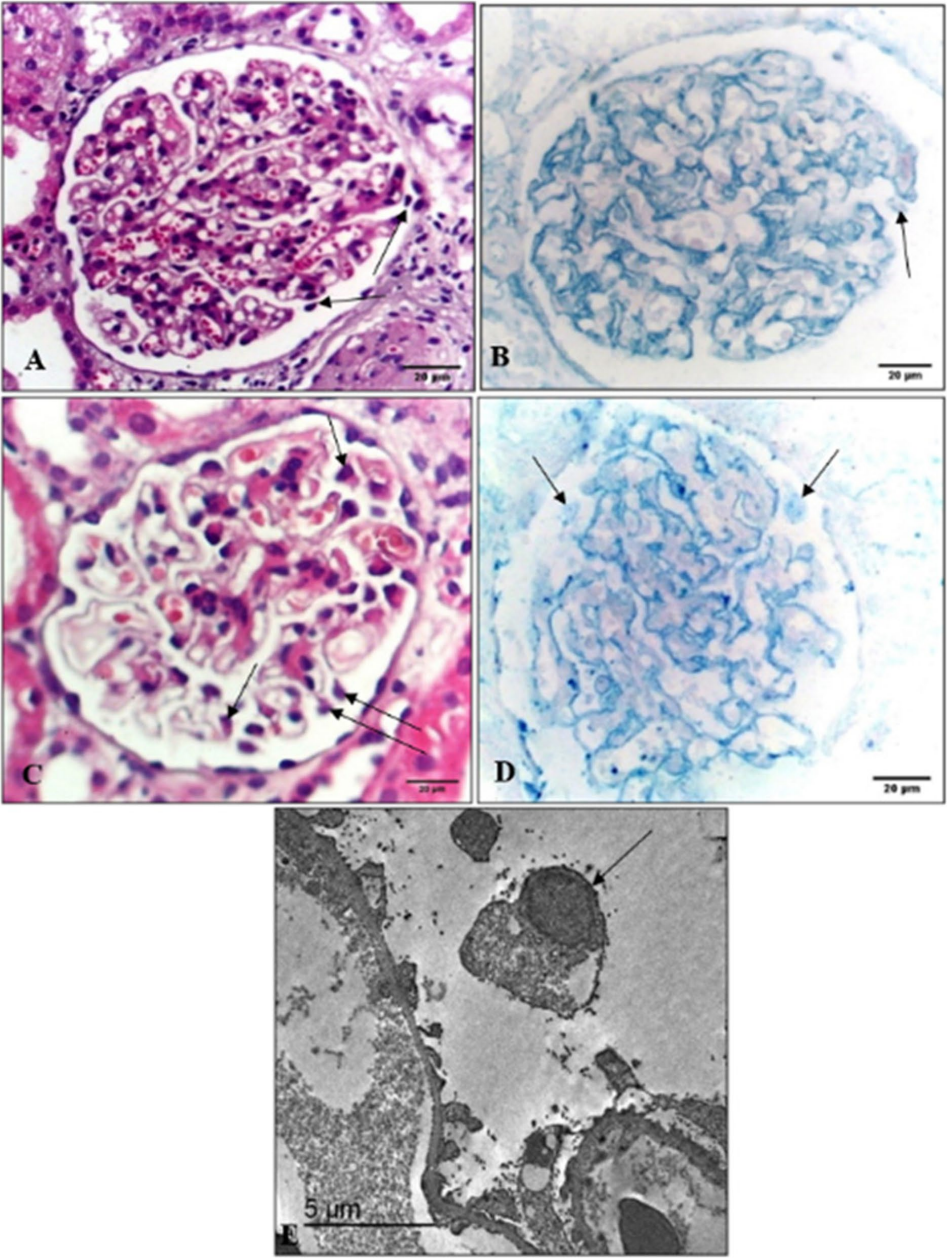
**A** Podoctyopathy with two detached podocytes (black arrows). H&E, 400 × . **B** Colloidal iron stain of the same case with detached podocyte highlighted by colloidal iron stain (black arrows), 400 × .** C** Case of podocytopathy with > three detached podocytes/glomerulus (black arrows). H&E, 400 × . **D** Colloidal iron stain of the same case with two detached podocytes in the Bowman’s space of the glomerulus (black arrows), 400 × . **E** EM examination of the same case shows focal fusion of foot processes of podocytes together with one detached podocyte (black arrow), 2,100 ×

**Fig. 3:**
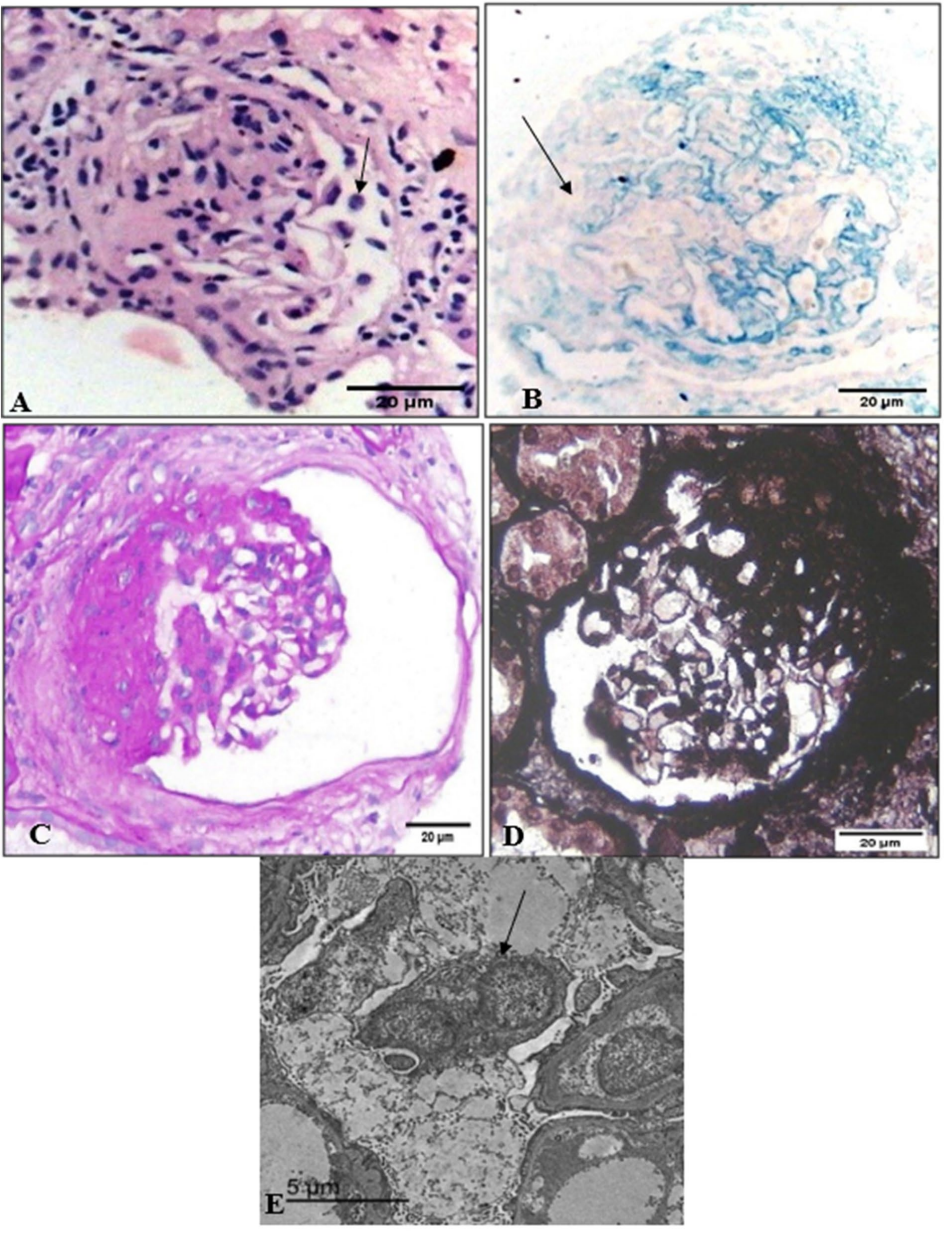
**A** FSGS, NOS with one detached podocyte (black arrows). H&E, 400 × .** B** Colloidal iron stain of the same glomerulus. The sclerosed area is devoid of podocyte coat and hence no podocyte coat of podocalyxin and no colloidal iron stain in this focus (black arrow). Colloidal iron, 400 × . **C** PAS stain of the same case of FSGS, NOS without observed detached podocytes, 400 × . **D** Silver stain of the same case of FSGS, NOS, 400 × . **E** EM examination of the same case showing diffuse fusion of foot processes of podocytes together with detached binucleated podocyte (black arrow), × 2,100

**Fig. 4:**
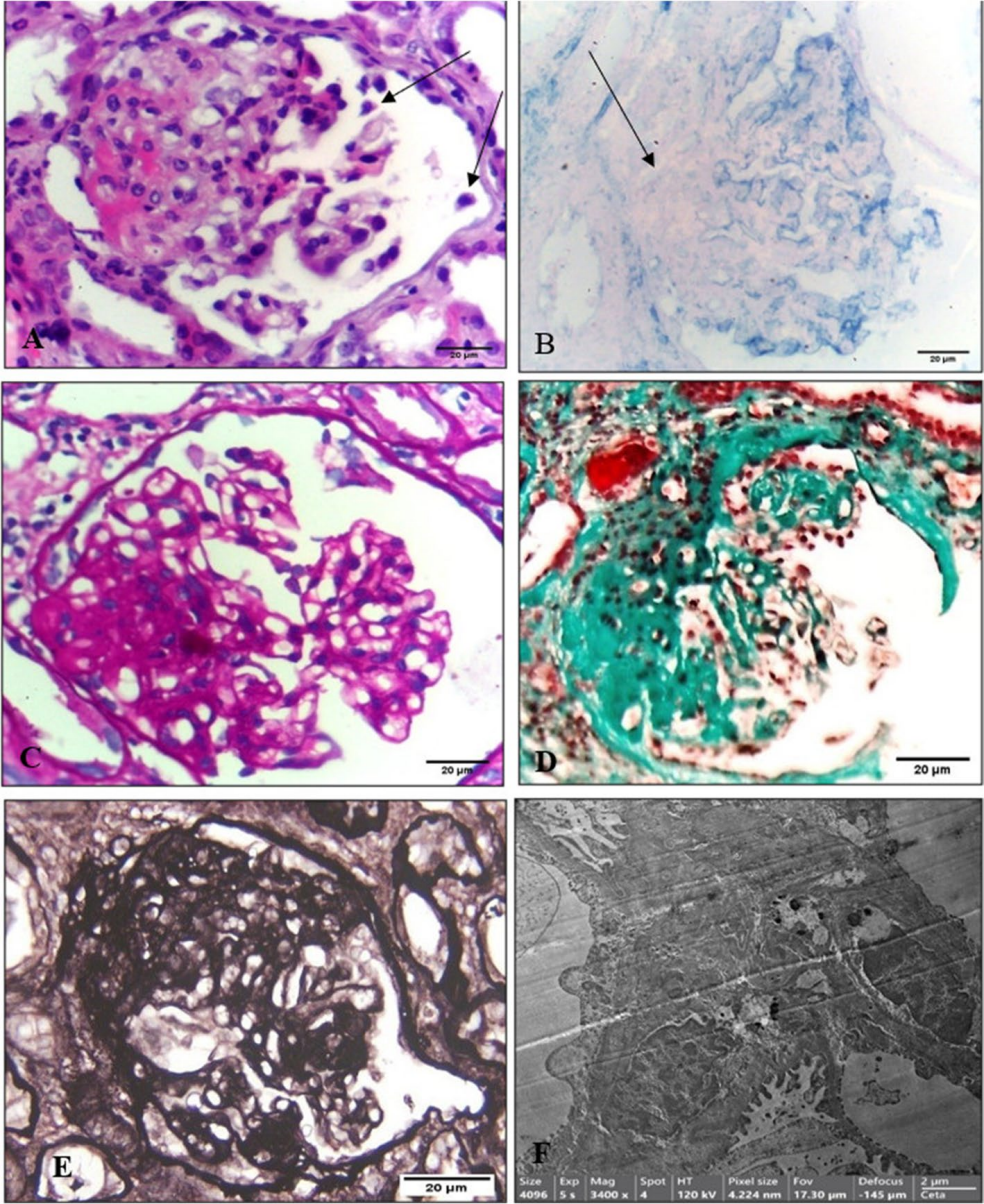
**A** FSGS, Hilar variant with detached podocytes in Bowman’s space (black arrows). H&E, 400 × . **B** Colloidal iron stain of the same case of FSGS, Hilar variant. The sclerosed area is devoid of podocyte coat and hence no podocyte coat of podocalyxin and no colloidal iron stain in this focus (black arrow), 400 × . **C** PAS stain of FSGS, Hilar variant, 400 × . **D** Masson trichrome stain of FSGS, Hilar variant, 400 × . **E** Silver stain of FSGS, Hilar variant, 400 × . **F** EM examination of FSGS, Hilar variant showing increased mesangial matrix

**Fig. 5:**
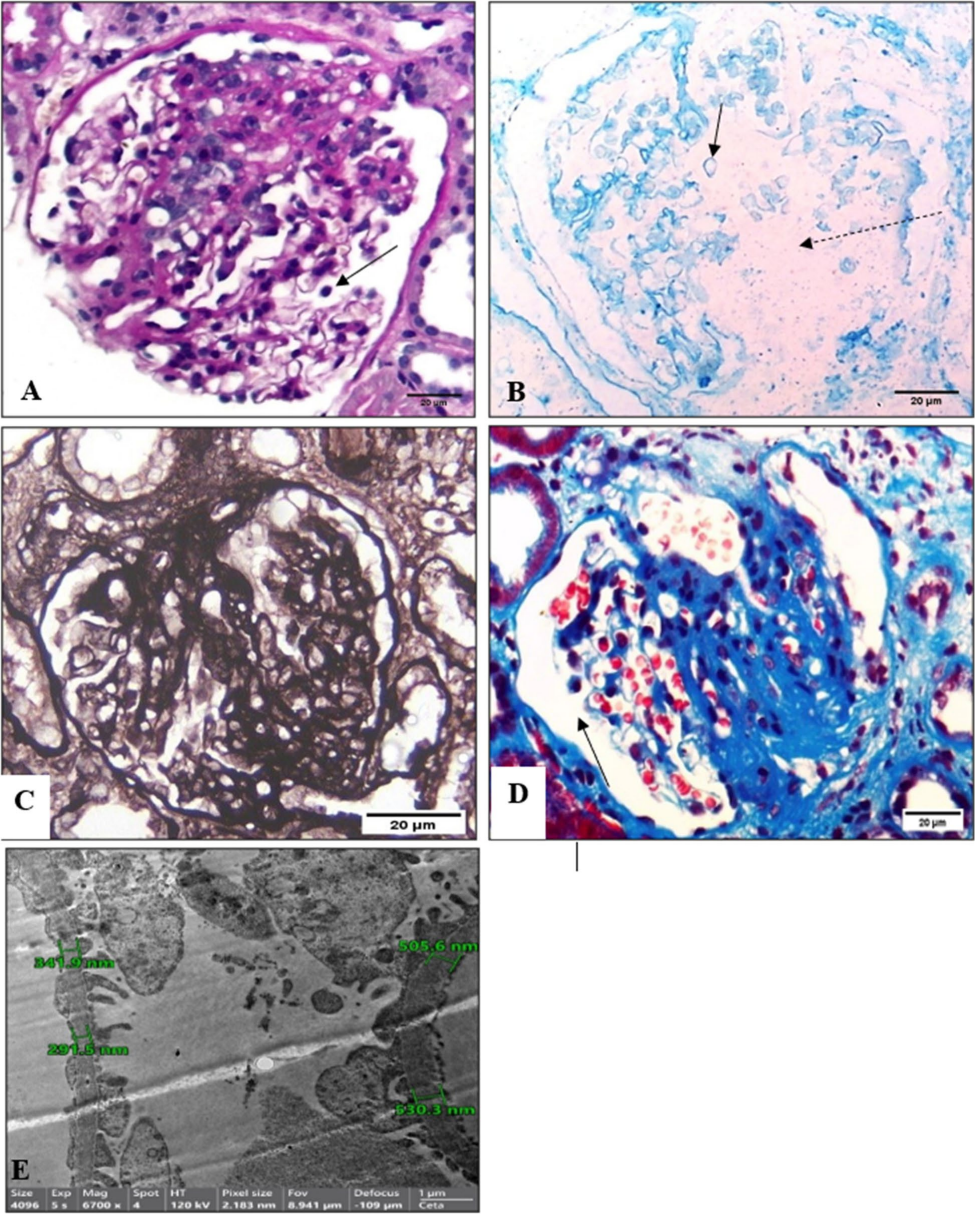
**A** PAS stain of FSGS, tip variant with focal area of sclerosis at tip region. There is one detached podocyte in the Bowman’s space (black arrow). PAS, 400 × . **B** Colloidal iron stain of the same case showing absent colloidal iron staining in sclerosed area (dashed arrow) with one detached podocyte/glomerulus (black arrow), 400 × . **C** Silver stain of FSGS, tip variant, 400 × . **D** FSGS, tip variant with one detached podocytes/glomerulus (black arrow), Masson trichrome stain, 200 × . **E** EM examination of FSGS, tip variant showing irregular thickness of basement membrane

Regarding the diagnosis of studied cases after EM, podocytopathy with detached podocytes is observed in 35 (52.24%) cases (Fig. 2), while FSGS formed 32 (47.76%) cases. In addition, FSGS cases demonstrated not otherwise specified, hilar, and tip variants that constituted 18 (17.14%), 8 (7.6%), and 6 (5.7%) cases, respectively (Figs. 3, 4, and 5).

The 11 (31.4%) cases diagnosed as podocytopathy with detached podocytes after EM examination showed false negative/no podocyte detachment by LM, while 16 (50%) of FSGS cases showed no podocyte detachment by LM. Score 1 podocyte detachment was observed in 22 (62.9%) of podoctyopathy with detached podocyte cases which showed a statistically significant difference (*p* ≤ 0.001). Scores 2 and 3, i.e., severe podocyte detachment were observed mainly in FSGS with 8 (25.0%) and 3 (9.4%) cases, respectively, in comparison to 0–1 cases with this score in the other 2 studied categories which demonstrated statistically significant difference (*p* ≤ 0.001). This finding indicates that severe podocyte detachment is significantly associated with the development of FSGS. This is depicted in Table 2. Regarding the evaluation of detached podocytes by EM, studied biopsies that showed detached podocytes by LM were confirmed by EM examination in about 95% of cases. When there was severe (score 3) podocyte detachment, EM confirmed podocyte detachment in 100% of the cases.

**Table 2:** Correlation between detached podocyte score by LM and final diagnosis after EM examination

**Detached podocytes score by LM**	**Final diagnosis after EM (total=67)**	FSGS (Total=32)	Test of significance
	Podocytopathy with detached podocytes (Total=35)		
Absent	11 (31.4%)	16 (50.0%)	MC *p*≤0.001*
1-2/glomerulus in <25% of total glomeruli	22 (62.9%) *	5 (15.6%)	
1-2/glomerulus in 25-50% or >50% of glomeruli	1 (2.9%)	8 (25.0%) *	
≥3/glom. in any percent of total glomeruli	1 (2.9%) *	3 (9.4%) *	

*MC* Monte Carlo test

*significant p≤0.05

There was a statistically significant difference between patient and control groups regarding mean PSA by colloidal iron staining with a *p* value of ≤ 0.001. The mean PSA among patient groups was around 17%, but it was higher among the control group which was noticed at around 23% as depicted in Table 3.

**Table 3:** Mean percent of colloidal iron staining among patients and normal groups

Mean percent-stained area (PSA) of glomeruli	**Patients group (no=67)**	**Control group** **(no=10)**	**Test of significance**	***p*** value
Mean ± SD Min-Max	17.61± 3.89 4.18- 30.47	23.61± 2.18 20.44- 27.46	t=10.24	≤0.001*

*t *independent t test

*significant *p* ≤0.05

There was detected a statistically significant difference between control cases, and each podocytopathy with detached podocytes and FSGS groups regarding mean PSA (*p* ≤ 0.001) which was 23.61% in the control group, got lowered to 18.95 ± 3.18 in podocytopathy with detached podocytes, but it was evidently lowered in FSGS to 14.28%. In addition, there was a statistically significant difference between FSGS patients and both podocytopathy with detached podocytes groups regarding mean PSA (*p* ≤ 0.001). Regarding the number of glomeruli with low PSA, there was also a statistically significant difference between FSGS patients and podocytopathy with detached podocytes groups (*p* ≤ 0.001) with a median number of 3 glomeruli in the first group as opposing to 6 glomeruli in the FSGS group as depicted in Table 4.

**Table 4:** Correlation between E/M diagnosis and mean percent-stained area (PSA)

	**Control group** **(total = 10)**	**Podocytopathy with detached podocytes** **(total = 35)**	**FSGS** **(total = 32)**	**Test of significance**
Total number of glomeruli/biopsy (mean ± SD)	51	16.67 ± 6.51	15.53 ± 6.12	*F* = 0.33 *p* = 0.718
Number of glomeruli with low percent of stained pixels/biopsy: median (Min–Max)	0 a	3 (0–21) b	6 (1–12) ab	KW = 25.65 *p* ≤ 0.001*
Mean percent-stained area (PSA) of glomeruli: mean ± SD	23.61 ± 2.18 ab	18.95 ± 3.18 a	14.28 ± 4.05 b	*F* = 24.3 *p* ≤ 0.001*

*F* ANOVA test, *KW* Kruskil-Wallis test, *ab* similar letters indicate a significant difference between groups

^*^Significant *p* ≤ 0.05

Regarding the ROC curve for prediction of the development of FSGS, it demonstrated a cutoff point of PSA < 16.08%, is mostly consistent with the FSGS category with accuracy of 86.7%, sensitivity of 75%, and specificity of 91.8%. The positive predictive value was 80 while the negative predictive value was 89.

**Fig. 6:**
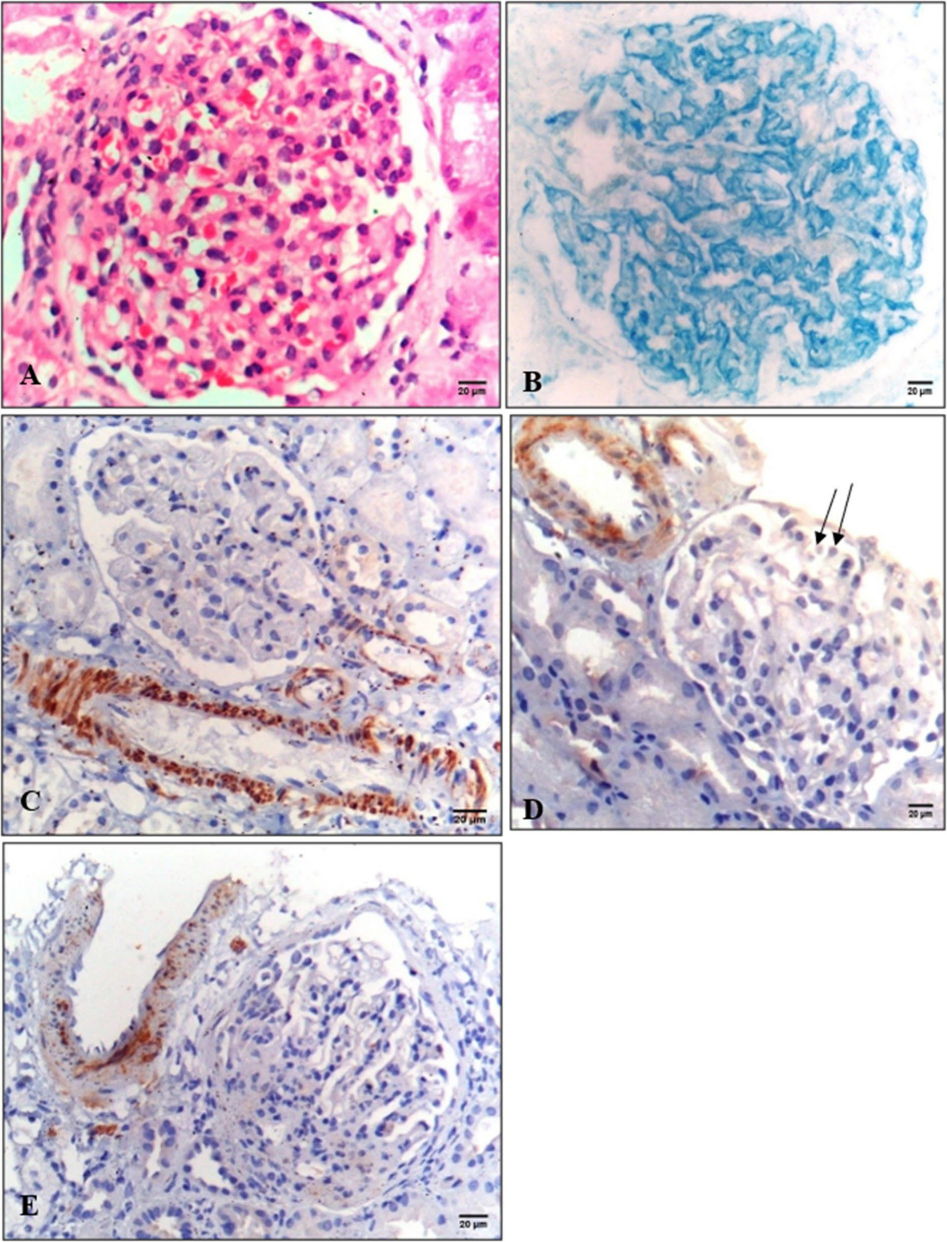
**A** Normal glomerulus, H&E, 400 × . **B** Colloidal iron stain of the same glomerulus with no detached podocytes/glomerulus, 400 × . **C** Immunoproxidase stain for desmin, control case, 200 × .** D** Podocytopathy with two detached podocytes/glomerulus (black arrows) with negative desmin immunoperoxidase stain, 200 × . **E** FSGS, perihilar variant with negative desmin immunoperoxidase stain, 200 × . Tissue control is smooth muscle fibers of interstitial vascular spaces

Immune staining with anti-desmin antibody revealed a negative reaction in podocytes with positive internal control for smooth muscle fibers in blood vessel walls in examined biopsies both for control and patient cases (Fig. 6).

## Discussion

Steroid-resistant nephrotic syndrome (SRNS) is one of the most common and perplexing clinical situations in nephrology practice where the patient has undergone renal biopsy for identification of the underlying pathology due to weak patient’s response to steroid therapy or the physician cannot withdraw steroids [2, 25–27]. A renal biopsy without thickening of the glomerular basement membrane or proliferation of mesangial cells, or mesangial thickening can be diagnosed as no abnormality detected by LM [28].

It has been postulated that podocytopathy plays a pivotal role in the pathogenesis of different glomerulonephritis patterns and it has turned out to be crucial to comprehensively describe abnormalities affecting podocytes in both LM and EM examination reports [29, 30]. One of the main limitations of the available podocytopathy studies on the web was the relatively small number of patients who underwent EM examination [31, 32]. If we can examine podocytopathy by LM, this might open the gate for research studies on a larger sample size [33, 34].

This study was designed to evaluate the advantage of LM in recognizing detached podocytes and subsequently podocytopenia. This study would introduce a novel, low-cost, and simple method for an in-depth evaluation of renal biopsies in patients presenting with SRNS. In the current study, we compared the patient group formed of 67 cases with the clinical diagnosis of SRNS and signed out after EM as FSGS and podocytopathy with detached podocytes to a control group formed of 10 cases obtained from surgical nephrectomy specimens. The patient group was predominantly of pediatric age category and male gender forming 64.8% and 72.4% of cases respectively. This was in keeping with a study conducted by Sobh et al. [35] who reported the same finding of the predominant pediatric age group forming 58% of the total examined population.

To evaluate the accuracy of LM as a tool for assessing podocytopenia, we needed to correlate the LM results to another reliable technique. Thus, we correlated LM to EM results [36]. Podocytes appear as small rounded dark nuclei with eosinophilic cytoplasm floating in the Bowman’s space and separated from the basement membrane [37]. Morphological features of podocytes are distinct from parietal epithelial cells, which are flat and not rounded [28, 38].

In the current study, we observed false-negative 11 (31.4%) cases without podocyte detachment by LM but were confirmed to have podocytopathy and podocyte detachment after EM examination. This is in contrast to what was reported by Sobh et al. [35] who found false negative results in 34/63 (54%) cases with no LM detachment but were confirmed after EM to have podocytopathy. This can be explained by the fact that EM magnification power can examine the sample thoroughly, thus revealing any podocyte injury, binucleation, or detachment that was not evident during LM examination.

The main task of this research was to establish a method for highlighting the podocyte coat of the glomerular basement membrane. A helpful approach to evaluate podocytopenia is by a quantitative assessment of the residual podocytes via a special podocyte stain; that is colloidal iron stain. This stain is based on the fact that podocytes are cells that are unique for their mucin coat of podocalyxin which binds to iron upon the first step of colloidal iron staining [20]. Then, the bound iron molecules are stained blue upon the second step of the staining [39]. In contrast to Weibel and Gomez’s method and Venkatareddy et al.’s methods [40], they tried to count the near absolute number of podocytes/each glomerulus. In the present study, we adopted that we do not have to do that. We suggested that it is better to know the percent rather than the actual number of podocyte cells in relation to the whole glomerular tuft surface area which is the percent of stained area by colloidal iron stain (PSA). PSA is calculated objectively by ImageJ analysis through a recorded macro that can obtain the results in a few seconds [22, 23].

By calculating the mean PSA for each case, we found that this convenient rapid, pathologist-friendly technique has a good positive diagnostic value in determining the degree of podocytopenia, predicting the pattern of podocyte injury, and helping to predict the resistance to steroid therapy. Sobh et al. [35] performed their study on control renal biopsies collected from radical nephrectomy specimens obtained from adult population and found that the mean PSA for their cases was 24% as a reference value for their control group sample [24]. Similarly, we found that the mean PSA of our control cases was 23.61 ± 2.18%, while the PSA of cases diagnosed as podocytopathy with detached podocytes showed a mean PSA of lower values (18.95 ± 3.18%). It was evidently lowered in FSGS to 14.28%. In the current study, we observed a statistically significant difference between the PSA of control cases and both podocytopathy with detached podocytes and FSGS categories (*p* ≤ 0.001). This finding was in keeping with what Sobh et al. [35] also concluded in their study. In podocytopathy with detached podocytes and hence FSGS categories, this could be attributed to that detached podocytes markedly decreased the surface area covered by colloidal iron stain and thus demonstrated an evident decrease in the PSA calculated by the ImageJ program.

Regarding the ROC curve for prediction of the development of FSGS, it demonstrated a cutoff point of PSA < 16.08%, which is mostly consistent with the FSGS category with a lower sensitivity of 75% and good specificity of over 90%. This is lower than the cutoff point stated by the study performed by Sobh et al. [24] who reported a cutoff point of 20.56% for diagnosing podocytopenia.

Desmin immunoperoxidase stain was done for all cases. None of the cases showed convincing positivity for the stain. In contrast to this study, a study conducted by Asakawa et al. [41] demonstrated that experimental hyperuricemia induced by uricase inhibition resulted in podocyte injury. This was confirmed by an increase in desmin expression in the injured podocytes. Also, another study assumed that the increase of desmin in glomerular epithelial cells was associated with glomerular epithelial damage in an experiment done to examine the features of aminonucleoside nephrosis [42]. Additionally, desmin immunohistochemistry revealed an obvious increase in desmin immunoreactivity in diabetic rats. Despite that, Losartan treatment displayed a decrease in desmin immunoreactivity in the same experiment [43]. However, similarly to the current study, desmin was not expressed in the early stages of kidney growth, infant and adult kidneys, and proliferative and non-proliferative glomerulonephritis in research done to demonstrate the expression of cytoskeletal proteins in glomerular epithelial cells [44].

The current study has some limitations. First, limited resources have contributed to the deficiency of genetic analysis for cases enrolled in this study. Determining the genetic background would help for better classification and recognition of disease etiologies [45, 46]. Second, researchers would conduct this study on a bigger number of cases with available EM examination reports. We recommend that upcoming studies may prospectively investigate the highlighting of podocytes for one of the specific podocyte markers like podocin, WT-1, synaptopodin antibody, podocalyxin, and nephrin [6]. Also, assessment of features of podocytopathy, hypertrophy, pyroptosis, apoptosis, necroptosis, mitotic catastrophe, and vacuolization can be done by LM examination with deceptively unremarkable glomeruli.

## Conclusion

LM detection of detached podocytes is a crucial finding suggesting the presence of FSGS. Standardized reporting of detached or decreased podocyte cells is becoming mandatory and strongly advised as it helps to be able to differentiate MCD from early FSGS. Also, they have a high positive predictive value for the expected EM picture of the case and that is the main recommendation of this study.

## Data Availability

No datasets were generated or analysed during the current study.

## References

[1] Singh L, Singh G, Dinda AK. Understanding podocytopathy and its relevance to clinical nephrology. Indian journal of nephrology. 2015;25(1):1–7. 25684864 10.4103/0971-4065.134531 PMC4323905

[2] Wang X, Zhao J, Li Y, et al. Epigenetics and endoplasmic reticulum in podocytopathy during diabetic nephropathy progression. Front Immunol. 2022;13: 1090989. 36618403 10.3389/fimmu.2022.1090989 PMC9813850

[3] Trimarchi H, Coppo R. Podocytopathy in the mesangial proliferative immunoglobulin A nephropathy: new insights into the mechanisms of damage and progression. Nephrol Dial Transplant. 2019;34(8):1280–5. 30698804 10.1093/ndt/gfy413

[4] Kostovska I, Trajkovska KT, Topuzovska S, et al. Nephrinuria and podocytopathies. Adv Clin Chem. 2022;108:1–36. 35659057 10.1016/bs.acc.2021.08.001

[5] Kostovska I, Tosheska Trajkovska K, Kostovski O, et al. Urinary nephrin and podocalyxin levels as predictors of pre-eclampsia in high-risk pregnant women. Folia Med. 2021;63(6):948–57. 10.3897/folmed.63.e60055 35851239

[6] Kandasamy Y, Smith R, Lumbers ER, et al. Nephrin - a biomarker of early glomerular injury. Biomarker research. 2014;2: 21. 25789166 10.1186/2050-7771-2-21 PMC4363192

[7] Banaszak B, Banaszak P. The increasing incidence of initial steroid resistance in childhood nephrotic syndrome. Pediatr Nephrol. 2012;27(6):927–32. 22231438 10.1007/s00467-011-2083-7 PMC3337414

[8] Uwaezuoke SN. The role of novel biomarkers in childhood idiopathic nephrotic syndrome: a narrative review of published evidence. Int J Nephrol Renov Dis. 2017;10:123–8. 10.2147/IJNRD.S131869 PMC5459980 28615961

[9] Sethna CB, Gipson DS. Treatment of FSGS in children. Adv Chronic Kidney Dis. 2014;21(2):194–9. 24602468 10.1053/j.ackd.2014.01.010

[10] Bai J, Yin X, Li J, et al. Incidence, risk factors, and outcomes of recurrent focal segmental glomerulosclerosis in pediatric kidney transplant recipients: a systematic review and meta-analysis. Clin Transplant. 2023;37(11): e15119. 37725070 10.1111/ctr.15119

[11] Shabaka A, Tato Ribera A, Fernández-Juárez G. Focal segmental glomerulosclerosis: state-of-the-art and clinical perspective. Nephron. 2020;144(9):413–27. 32721952 10.1159/000508099

[12] McGrogan A, Franssen CF, de Vries CS. The incidence of primary glomerulonephritis worldwide: a systematic review of the literature. Nephrol Dial Transplant. 2011;26(2):414–30. 21068142 10.1093/ndt/gfq665

[13] Sim JJ, Batech M, Hever A, et al. Distribution of biopsy-proven presumed primary glomerulonephropathies in 20002011 among a racially and ethnically diverse US population. Am J Kidney Dis. 2016;68(4):533–44. 27138468 10.1053/j.ajkd.2016.03.416

[14] Rosenberg AZ, Kopp JB. Focal segmental glomerulosclerosis. Clinical journal of the American Society of Nephrology : CJASN. 2017;12(3):502–17. 28242845 10.2215/CJN.05960616 PMC5338705

[15] Harshman LA, Bartosh S, Engen RM. Focal segmental glomerulosclerosis: risk for recurrence and interventions to optimize outcomes following recurrence. Pediatr Transplant. 2022;26(6): e14307. 35587003 10.1111/petr.14307

[16] Sinha A, Bagga A. Nephrotic syndrome. Indian J Pediatr. 2012;79(8):1045–55. 22644544 10.1007/s12098-012-0776-y

[17] Erichsen L, Thimm C, Bohndorf M, et al. Activation of the renin-angiotensin system disrupts the cytoskeletal architecture of human urine-derived podocytes. Cells. 2022;11(7):1095. 35406662 10.3390/cells11071095 PMC8997628

[18] Barisoni L, Schnaper HW, Kopp JB. A proposed taxonomy for the podocytopathies: a reassessment of the primary nephrotic diseases. Clinical journal of the American Society of Nephrology: CJASN. 2007;2(3):529–42. 17699461 10.2215/CJN.04121206

[19] Tickoo SK, Amin MB, Zarbo RJ. Colloidal iron staining in renal epithelial neoplasms, including chromophobe renal cell carcinoma: emphasis on technique and patterns of staining. Am J Surg Pathol. 1998;22(4):419–24. 9537468 10.1097/00000478-199804000-00005

[20] Hussein AM, Eldosoky M, Handhle A, et al. Effects of long-acting erythropoietin analog darbepoetin-α on adriamycin-induced chronic nephropathy. Int Urol Nephrol. 2016;48(2):287–97. 26660954 10.1007/s11255-015-1171-1

[21] Elkholy IAGAMI, Mohammad MA, Zayed DHM, Elkalla HMHR, Elhilali AMA, Refat S. Pattern of expression of Delta-like ligand 4 (DLL-4) in patients with pancreatic ductal and ampullary adenocarcinomas and its clinicopathological prognostic implications. IP J Diagn Pathol Oncol. 2020;5(3):257–66.

[22] Barisoni L, Lafata KJ, Hewitt SM, et al. Digital pathology and computational image analysis in nephropathology. Nat Rev Nephrol. 2020;16(11):669–85. 32848206 10.1038/s41581-020-0321-6 PMC7447970

[23] Cazzaniga G, Rossi M, Eccher A, et al. Time for a full digital approach in nephropathology: a systematic review of current artificial intelligence applications and future directions. J Nephrol. 2024;37(1):65–76. 37768550 10.1007/s40620-023-01775-w PMC10920416

[24] Moustafa FE, Sobh MM. How to quantify podocytopenia in the clinical practice? A new step in computational renal pathology. Available at SSRN: 10.2139/ssrn.3987062.

[25] Warejko JK, Tan W, Daga A, et al. Whole exome sequencing of patients with steroid-resistant nephrotic syndrome. Clinical journal of the American Society of Nephrology : CJASN. 2018;13(1):53–62. 29127259 10.2215/CJN.04120417 PMC5753307

[26] Chan EY, Yap DY, Colucci M, et al. Use of rituximab in childhood idiopathic nephrotic syndrome. Clinical journal of the American Society of Nephrology: CJASN. 2023;18(4):533–48. 36456193 10.2215/CJN.08570722 PMC10103321

[27] Carter SA, Mistry S, Fitzpatrick J, et al. Prediction of short- and long-term outcomes in childhood nephrotic syndrome. Kidney international reports. 2020;5(4):426–34. 32280840 10.1016/j.ekir.2019.12.015 PMC7136435

[28] Gomez AC, Gibson KL, Seethapathy H. Minimal change disease. Advances in kidney disease and health. 2024;31(4):267–74. 39084752 10.1053/j.akdh.2024.02.002

[29] Chen D, Hu W. Lupus podocytopathy: a distinct entity of lupus nephritis. J Nephrol. 2018;31(5):629–34. 29270846 10.1007/s40620-017-0463-1

[30] Ahmadian E, Eftekhari A, Atakishizada S, et al. Podocytopathy: the role of actin cytoskeleton. Biomed Pharmacother. 2022;156:113920. 36411613 10.1016/j.biopha.2022.113920

[31] AlYousef A, AlSahow A, AlHelal B, et al. Glomerulonephritis histopathological pattern change. BMC Nephrol. 2020;21(1):186. 32423387 10.1186/s12882-020-01836-3 PMC7236312

[32] Ibrahim S, Fayed A, Fadda S, et al. A five-year analysis of the incidence of glomerulonephritis at Cairo University Hospital-Egypt. Saudi journal of kidney diseases and transplantation : an official publication of the Saudi Center for Organ Transplantation, Saudi Arabia. 2012;23(4):866–70. 22805412 10.4103/1319-2442.98191

[33] Oliva-Damaso N, Payan J, Oliva-Damaso E, et al. Lupus podocytopathy: an overview. Adv Chronic Kidney Dis. 2019;26(5):369–75. 31733721 10.1053/j.ackd.2019.08.011 PMC8405037

[34] Maas RJ, Nijenhuis T, van der Vlag J. Minimal change disease: more than a podocytopathy? Kidney international reports. 2022;7(4):675–7. 35497782 10.1016/j.ekir.2022.03.001 PMC9039900

[35] Sobh MM, El Kannishy G, Moustafa F, et al. Role of detached podocytes in differentiating between minimal change disease and early focal segmental glomerulosclerosis, can we rely on routine light microscopy? J Nephrol. 2022;35(9):2313–24. 36350562 10.1007/s40620-022-01456-0 PMC9700609

[36] Rovin BH, Caster DJ, Cattran DC, et al. Management and treatment of glomerular diseases (part 2): conclusions from a Kidney Disease: Improving Global Outcomes (KDIGO) controversies conference. Kidney Int. 2019;95(2):281–95. 30665569 10.1016/j.kint.2018.11.008

[37] Xia W, Deng J, Zhuang L, et al. Risk factors for acute kidney injury and kidney relapse in patients with lupus podocytopathy. Clinical kidney journal. 2024;17(6):sfae148. 38835511 10.1093/ckj/sfae148 PMC11145460

[38] Li ZH, Guo XY, Quan XY, et al. The role of parietal epithelial cells in the pathogenesis of podocytopathy. Front Physiol. 2022;13: 832772. 35360248 10.3389/fphys.2022.832772 PMC8963495

[39] Gupta N, Panigrahi A, Gupta N, et al. Macular corneal dystrophy with iridofundal coloboma in the same patient: a unique combination. BMJ case reports. 2024;17(5):e258786. 38719268 10.1136/bcr-2023-258786 PMC11085980

[40] Venkatareddy M, Wang S, Yang Y, et al. Estimating podocyte number and density using a single histologic section. J Am Soc Nephrol. 2014;25(5):1118–29. 24357669 10.1681/ASN.2013080859 PMC4005315

[41] Asakawa S, Shibata S, Morimoto C, et al. Podocyte injury and albuminuria in experimental hyperuricemic model rats. Oxid Med Cell Longev. 2017;2017: 3759153. 28337250 10.1155/2017/3759153 PMC5350416

[42] Yaoita E, Kawasaki K, Yamamoto T, et al. Variable expression of desmin in rat glomerular epithelial cells. Am J Pathol. 1990;136(4):899–908. 2183627 PMC1877632

[43] Kakimoto T, Okada K, Hirohashi Y, et al. Automated image analysis of a glomerular injury marker desmin in spontaneously diabetic Torii rats treated with losartan. J Endocrinol. 2014;222(1):43–51. 24781258 10.1530/JOE-14-0164

[44] Gonlusen G, Ergin M, Paydaş S, et al. The expression of cytoskeletal proteins (alpha-SMA, vimentin, desmin) in kidney tissue: a comparison of fetal, normal kidneys, and glomerulonephritis. Int Urol Nephrol. 2001;33(2):299–305. 12092643 10.1023/a:1015226426000

[45] Chatterjee R, Hoffman M, Cliften P, et al. Targeted exome sequencing integrated with clinicopathological information reveals novel and rare mutations in atypical, suspected and unknown cases of Alport syndrome or proteinuria. PLoS One. 2013;8(10): e76360. 24130771 10.1371/journal.pone.0076360 PMC3794937

[46] Büscher AK, Kranz B, Büscher R, et al. Immunosuppression and renal outcome in congenital and pediatric steroid-resistant nephrotic syndrome. Clinical journal of the American Society of Nephrology: CJASN. 2010;5(11):2075–84. 20798252 10.2215/CJN.01190210 PMC3001773

